# The paradox of lumbopelvic alignment in anterolateral and posterolateral phenotypes of symptomatic hip dysplasia

**DOI:** 10.1002/jeo2.70123

**Published:** 2024-12-30

**Authors:** Marco Haertlé, Malte Lübbecke, Nils Becker, Henning Windhagen, Quentin Karisch, Sufian S. Ahmad

**Affiliations:** ^1^ Department of Orthopaedic Surgery Hannover Medical School Hannover Germany

**Keywords:** developmental dysplasia of the hip, pelvic tilt, phenotypes of hip dysplasia, radiographic analysis, sagittal lumbopelvic alignment

## Abstract

**Purpose:**

The relationship between sagittal lumbopelvic alignment and the bony pathomorphology of hip dysplasia is currently at the forefront of clinical and scientific interest. The aim of this study was to determine whether there is a compensatory lumbopelvic aspect associated with the two major acetabular phenotypes in dysplastic hips.

**Methods:**

From September 2022 to March 2024, a total of 145 patients with symptomatic bilateral hip dysplasia were included in the study. Hips were categorized into either anterolateral or posterolateral morphologies based on anteroposterior pelvic x‐rays. Additionally, the lumbopelvic sagittal alignment was determined radiographically. Furthermore, a multivariable linear regression analysis was conducted to assess the association of lumbopelvic sagittal alignment with additional independent factors.

**Results:**

Pelvic tilt (PT) significantly differed between the anterolateral and posterolateral phenotypes of hip dysplasia (16.84° ± 8.75° vs. 11.51° ± 6.63°, respectively; *p* < 0.001). Similar significant findings were observed for pelvic incidence (57.19° ± 12.96° vs. 50.75° ± 13.1°, respectively; *p* < 0.001). A PT of >14.5° was identified as the most likely factor associated with anterolateral dysplasia.

**Conclusions:**

The results of this study reveal a paradox in the hip–spine association in hip dysplasia. Contrary to previous theories, it seems that PT constitutes a component of the corresponding phenotype of dysplastic pathology, rather than functioning as a compensatory tilt.

**Level Evidence:**

Level III.

**Abbreviations**
AC: anterior wall coverage
AP: anteroposterior
DDH: developmental dysplasia of the hip
LCEA: lateral centre‐edge angle
PC: posterior wall coverage
PI: pelvic incidence
PT: pelvic tilt
ROC: receiver operating characteristic
SS: sacral slope

## INTRODUCTION

The understanding and treatment of developmental dysplasia of the hip (DDH) have evolved over recent years [9, 29]. The standard method for diagnosis is the conventional anteroposterior (AP) radiograph of the pelvis, which provides a visual assessment of the lateral, anterior and posterior coverage of the femoral head [22]. It has been recognized that the complex three‐dimensional pathology of acetabular under coverage could involve the anterior wall, the posterior wall, the superior roof or any specific combination of features [2, 7, 20]. This has led clinicians to adapt classification systems that help distinguish between the different phenotypes of hip dysplasia [2, 28]. The importance of such concepts is growing due to the necessity for a thorough understanding of the unique morphology in order to establish individualized treatment plans [1].

Alongside the morphological characteristics of hip dysplasia, the impact of sagittal lumbopelvic alignment on the condition has gained significant interest. This is primarily due to the questions arising from the likelihood of anterior acetabular coverage being influenced by flexion of the pelvis and lumbar lordosis. Whether this pelvic tilt (PT) is functional or morphological remains unclear [19, 21, 27]. A potential functional component of PT could significantly impact treatment protocols.

This study aimed to investigate the association between sagittal lumbopelvic alignment and the phenotype of dysplasia in patients presenting with symptomatic hip dysplasia. It was hypothesized that a decreased PT would be linked with bilateral anterolateral dysplasia, serving as a compensatory mechanism to address the inadequate anterior coverage of the femoral head.

## METHODS

**Figure 1:**
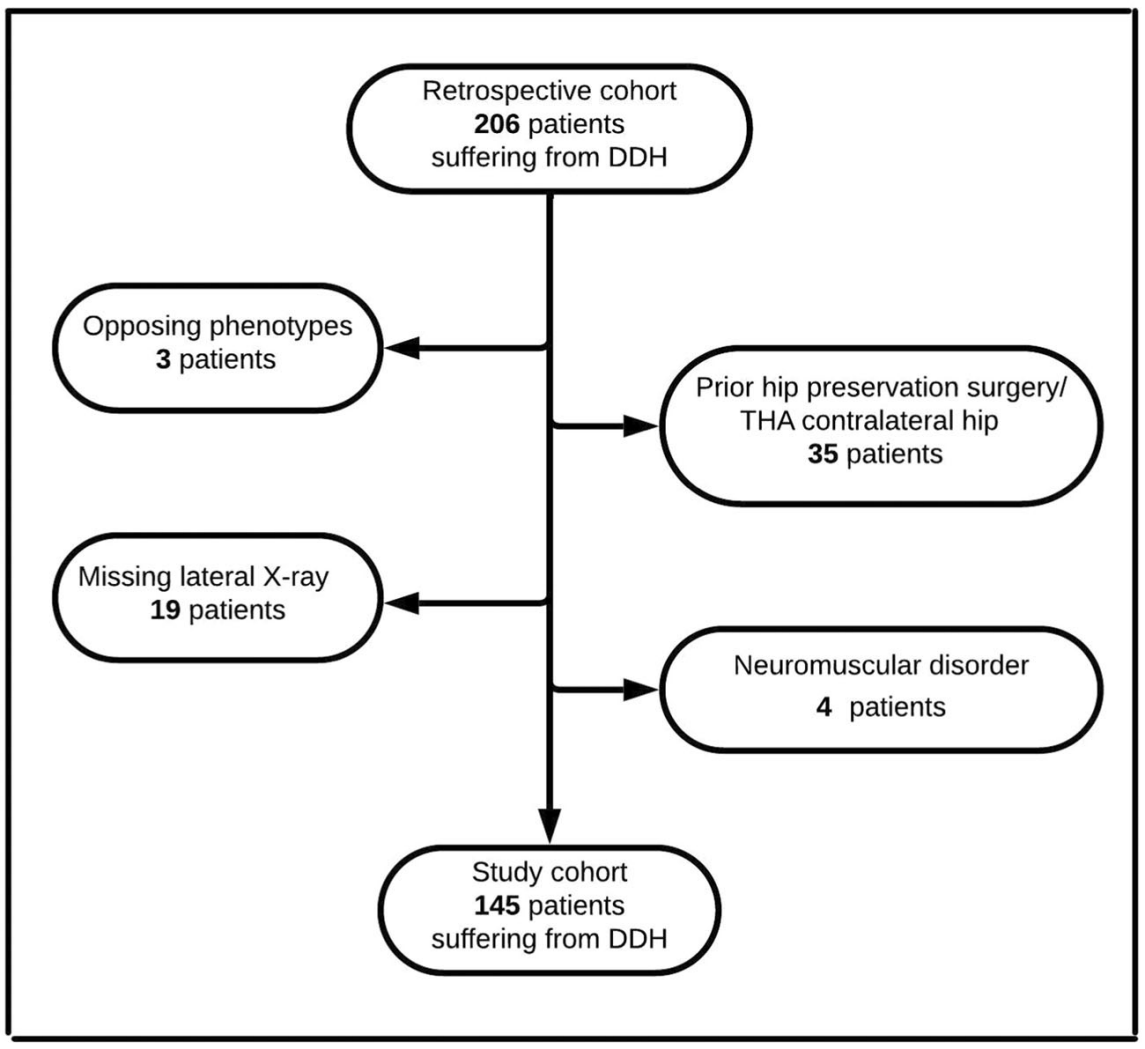
The flowchart displays the excluded patients from the investigated study population.

Patients presenting symptomatic bilateral hip dysplasia between September 2022 and March 2024 were considered eligible for inclusion in the study. Patients were enrolled if their pain was clinically attributed to the pathology of hip dysplasia, defined as having a lateral centre‐edge angle (LCEA) of <25° on a supine AP pelvic x‐ray. Exclusion criteria were a unilateral phenotype of hip dysplasia, previous hip surgery on the ipsilateral or contralateral hip, degenerative disease of the hip (Tönnis > 1), previous spine surgery, age > 45 years that may be associated with degenerative stiffness, missing radiographs and hip dysplasia in association with neuromuscular comorbidity (Figure 1).

From an initial cohort of 206 patients with symptomatic hip dysplasia, 145 were included in the study (Figure 1).

### Radiographic evaluation and determination of dysplasia phenotype

Following the standard clinical assessment, all patients underwent radiographic evaluation, including an AP pelvic x‐ray in the supine position, and a standing lateral pelvic radiograph capturing both femoral heads and the fifth lumbar vertebra [18]. Radiographic morphology of the hips was evaluated by assessing both quantitative and qualitative parameters, including the LCEA, acetabular index, extrusion index, anterior wall coverage (AC), posterior wall coverage (PC) and rotation of the hemipelvis.

The sagittal lumbopelvic alignment was assessed by measuring sagittal slope (SS), PT and pelvic incidence (PI). The mean values for lumbopelvic sagittal alignment have been well studied and defined. For SS, which is defined as the angle between the tangent to the apex of the sacrum and a horizontal line, the average values in the normal population range between 35° and 45° [10]. PT, which is a parameter for pelvic mobility, was measured as the angle between a vertical line from the centre of the femoral heads and a connecting line between the centre of the femoral heads and the midpoint the sacral endplate. The mean value for PT in the normal population was reported to be 12°. In contrast, PI is a morphological measure of the pelvis that remains constant throughout life. PI is calculated as the sum of PT and SS. On average, the PI in the normal population measures 53° [3].

**Figure 2:**
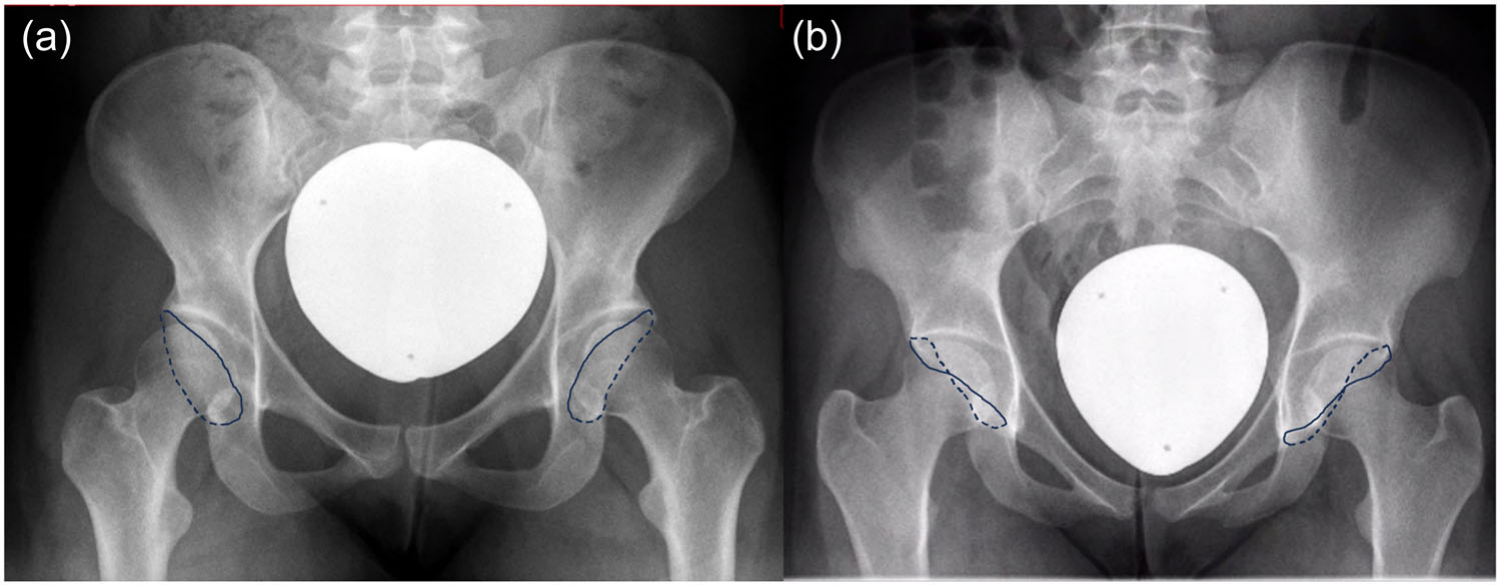
(a) The x‐ray image depicts an anterolateral dysplasia, characterized by reduced lateral coverage and reduced anterior wall coverage of the femoral head. Additionally, the iliac wings appear inwardly rotated. (b) In contrast, posterolateral dysplasia is characterized by an increased anterior coverage of the femoral head, accompanied by a concurrently reduced posterior head coverage and a pronounced crossover sign of the acetabular rims. Additionally, the iliac wings are markedly externally rotated, resulting in a visibly broader appearance of the hemipelvis. (Anterior acetabular rim = solid blue line, posterior acetabular rim = dashed blue line).

Hips were radiographically categorized into anterolateral or posterolateral dysplasia. The anterolateral phenotype was defined by a deficient lateral coverage (LCEA < 25°), a crossover‐index < 10%, AC of <25% and reduced external rotation of the iliac wing. Vice versa, the posterolateral phenotype was characterized by a crossover‐index > 10%, AC of >25%, posterior wall sign and increased external rotation of the iliac wing (Figure 2) [11]. The phenotyping of the hip joints was performed for both hips. The assessment of the phenotype was conducted independently by two hip preservation surgeons. In cases of nonconformity regarding the phenotype, a consensus decision was attained through consultation with a third hip surgeon.

### Variables and endpoints

The primary aim of this study was to determine the relationship between sagittal lumbopelvic alignment and the phenotype of acetabular dysplasia. Sagittal lumbopelvic alignment was defined using the parameters of SS, PT and PI. Patients were grouped according to the above described classification of the phenotype of hip dysplasia. Secondary variables included gender, LCEA (°) and disruption of the Shenton line.

### Statistical analysis

Data were tabulated and continuous variables were presented as mean ± standard deviation. Nominal variables were presented in binary format. Comparison between means was performed using analysis of variance (ANOVA). A multivariate linear regression model was used to identify factors associated with the phenotype of DDH which was defined as the output variable. Measurements for lumbopelvic alignment, LCEA and disruption of the Shenton line were defined as input variables. Disruption of the Shenton line and a PT >14,5° were included as nominal input factors. Reverse adjustment was subsequently performed. Receiver operating characteristic (ROC) analysis was performed to determine a cutoff value for PT for binary inclusion in the regression model. There were no missing data. A *p *< 0.05 was considered statistically significant. IBM SPSS software version 24 (IBM statistics,) and Prism 10 (GraphPad Software) were used for analysis.

### Ethical review committee statement

The study adhered to the principles outlined in the Declaration of Helsinki and received approval from the local Ethics Committee of the Hannover Medical School (code: 11329_BO_K_2024 date: 04.04.2024). The study was carried out at the Hannover Medical School, Department of Orthopaedic Surgery, Anna‐von‐Borriesstr. 1‐7, 30625 Hannover, Germany.

## RESULTS

**Figure 3:**
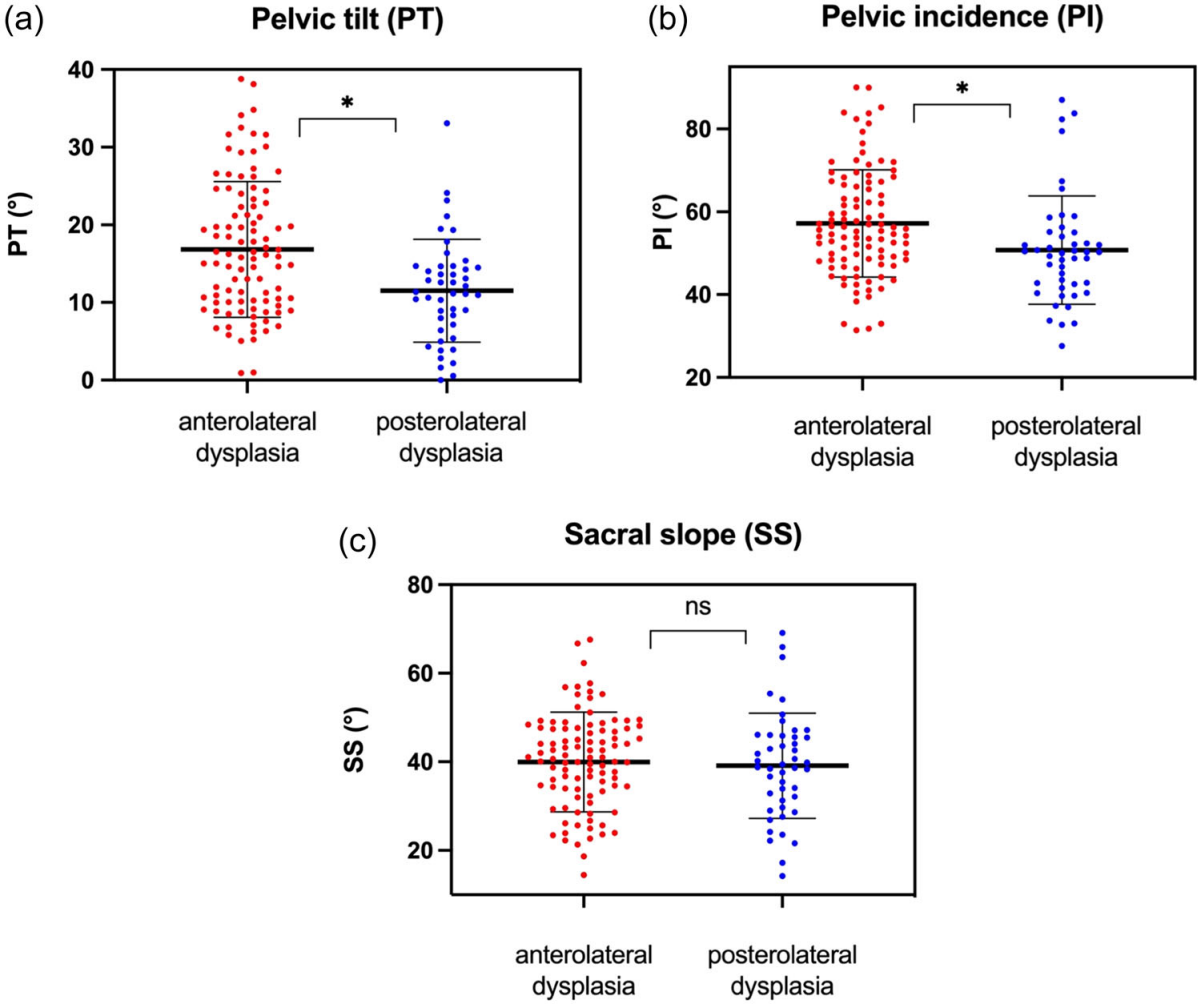
(a) Plot demonstrating that the reduced anterior coverage in dysplastic hips is associated significantly with an increase in pelvic tilt compared to dysplastic hips with diminished posterior acetabular coverage (16.84° ± 8.75° vs. 11.51° ± 6.63°, *p* = 0.0002). (b) The same relationship was shown in regard to pelvic incidence (57.19° ± 12.96° vs. 50.75° ± 13.1°, *p* = 0.0031). (c) Whereas no significant difference of sacral slope was evident when comparing anterolateral and posterolateral type of hip dysplasia (39.95° ± 11.27° vs. 39.13° ± 11.88°, *p* = 0.34).

A total of 145 patients with bilateral hip dysplasia were included in this study. Among these, 99 (68.28%) were categorized as anterolateral dysplasia (Table 1). The posterolateral dysplasia group exhibited a notably larger proportion of male patients compared to the anterolateral hip dysplasia cohort (*p* = 0.04) (Table 1). Patients presenting with an anterolateral phenotype of acetabular under‐coverage demonstrated a significantly increased PT in comparison to those with posterolateral dysplasia (Figure 3a). Additionally, PI was considerably higher in the anterolateral dysplasia group (Figure 3b). However, the sagittal slope (SS) showed no significant variations between the two dysplasia phenotypes (Figure 3c).

**Table 1:** Descriptive data ALD versus PLD anaesthesia.

Number of patients	ALD	PLD	*p* Value
Ø Age (years)	99/145 (68.28%)	46/145 (31.72%)	
Sex	29.51 ± 8.27	27.80 ± 6.48	0.10
Female	85/99 (85.86%)	32/46 (69.57%)	0.04
Body mass index (kg/m^2^)	14/99 (14.14%)	14/46 (30.43%)	
Radiographic data	24.98 ± 5.03	25.76 ± 4.51	0.19
LCEA (°)	15.48 ± 5.88	19.73 ± 7.41	<0.001
Pelvic tilt (°)	16.84 ± 8.75	11.51 ± 6.63	<0.001
Sacral slope (°)	39.95 ± 11.27	39.13 ± 11.88	0.34
Pelvic incidence (°)	57.19 ± 12.96	50.75 ± 13.1	0.003
Disrupted Shenton Line	13/99 (13.13%)	2/46 (4.35%)	0.19

Abbreviations: ALD, anterolateral dysplasia; LCEA, lateral centre‐edge angle; PLD, posterolateral dysplasia.

ROC analysis revealed that a PT > 14.5° was most strongly indicative of an anterolateral dysplastic phenotype with a sensitivity of 0.58 and 1‐specificity of 0.26.

Multivariable linear regression identified a PT of >14.5° as the only significant independent factor associated with anterolateral dysplasia (Table 2).

**Table 2:** Multivariable linear regression analysis demonstrating pelvic tilt as the only independent factor associated with the phenotype of hip dysplasia (anterolateral vs. posterolateral).

Dependent factor		Confidence interval			
Type of dysplasia (anterolateral vs. posterolateral)	Independent factor	Beta (*β*)	Regression coefficient (B)	Standard error	Sig
	Pelvic incidence	0.98	−0.03	0.02	0.16
	**Pelvic tilt > 14.5°**	**4.41**	**1.48**	**0.4**	**<0.001**
	Disrupted Shenton line	0.29	−1.25	0.81	0.11

## DISCUSSION

**Figure 4:**
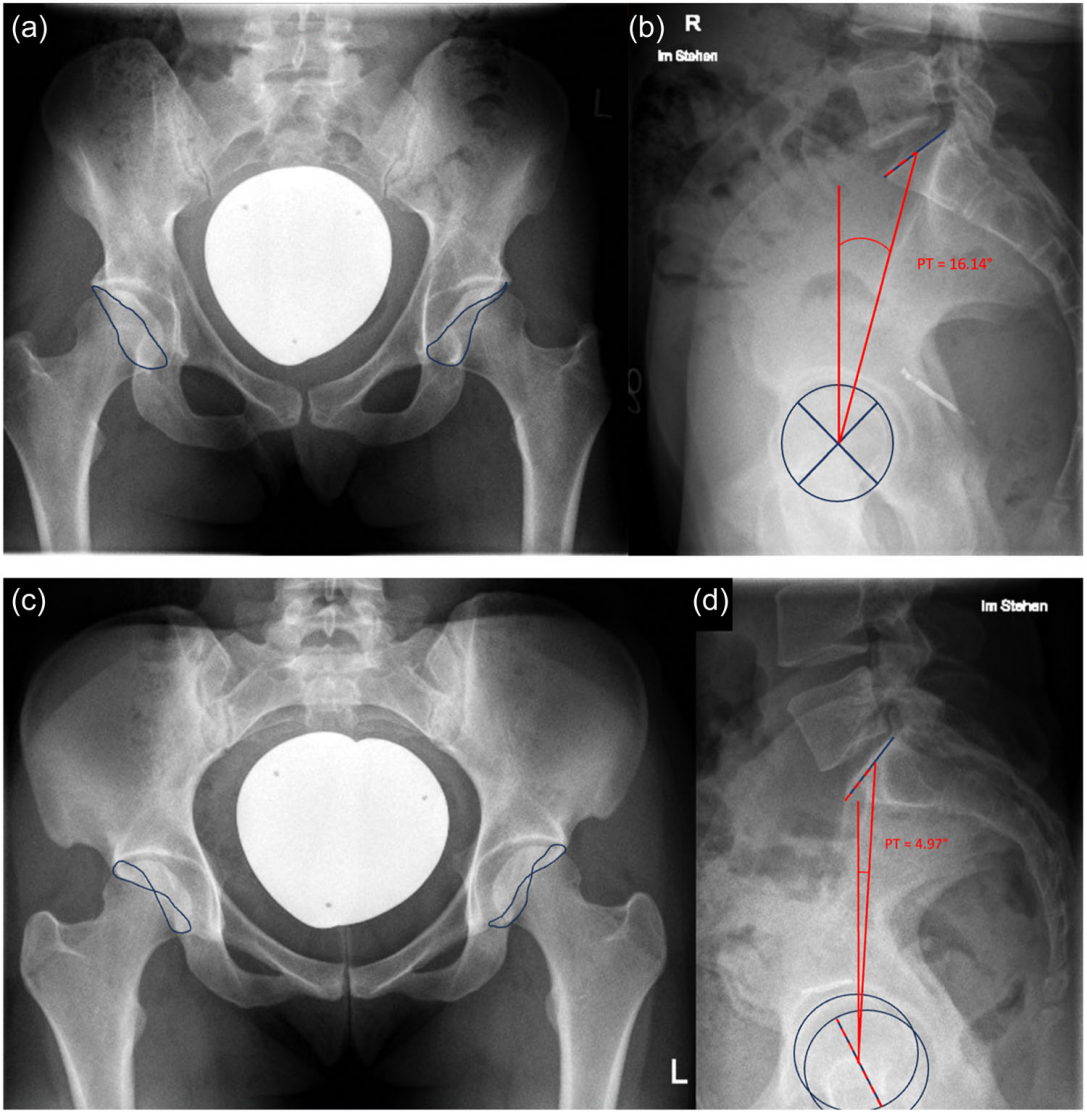
(a) A supine pelvic x‐ray in anteroposterior projection reveals bilateral anterolateral dysplasia. Alongside the reduced lateral acetabular coverage, there is also diminished anterior coverage. The lateral projection of this pelvic x‐ray in standing position exhibits a markedly increased pelvic tilt (b). In contrast, (c) depicts a radiographic projection of bilateral posterolateral dysplasia with a corresponding pronounced cross‐over sign. The associated lateral x‐ray reveals a significant anterior tilt of the sacrum, resulting in a reduced pelvic tilt (d). The continuous blue line represents the acetabular rim.

This study yielded two major findings. First, a significant link was established between the dysplastic acetabular phenotype and the sagittal alignment of the pelvis, as illustrated in Figure 4. Second, the findings reveal that a PT > 14.5° corresponds with an anterolateral dysplastic phenotype, implying that an increased PT could potentially worsen the issue of inadequate anterolateral coverage of the femoral head instead of compensating for it.

Lumbopelvic sagittal alignment is widely recognized as a dynamic component in hip joint pathologies [14, 17]. Several studies have shown that PT can temporarily change during the postoperative phase following hip preservation surgery [27]. It has also been observed that the degree of preoperative PT can affect the postoperative outcomes of dysplastic hips [23].

It has been proposed that alterations in PT may function as a compensatory mechanism inherent to hip morphology to improve femoral head coverage in DDH hips [6, 12]. This phenomenon has been particularly reported in arthritic hips that present with flexion contractures, accompanied by a correspondingly reduced PT [16, 26].

On the contrary, this study focused on patients with nonarthritic dysplastic hips, where hip flexion contracture is not a primary pathology, unlike in arthritic hips.

There has been an increasing tendency toward categorizing DDH into distinct phenotypes, including anterolateral and posterolateral, based on anterior and posterior wall coverage in association with rotation of the inferior hemipelvis [2, 15].

Studies that describe compensatory PT in patients with DDH have not explored the association between the specific DDH phenotypes and lumbopelvic alignment [5, 6]. Nevertheless, a number of studies have focused on assessing lumbopelvic alignment in retroverted hip morphology. Lerch et al. demonstrated significantly lower PT and PI values in hips with femoroacetabular impingement, compared to dysplastic hips [11]. Another analysis of 478 hips provided evidence for a significant association between acetabular retroversion and reduced PI, which is in agreement with our current findings [24].

Based on the current literature and the results of this study, there seems to be a paradox regarding the role of functional lumbopelvic alignment in dysplasia. The prevailing theory that PT compensated for dysplasia will need to be challenged in the future.

The fact that sagittal alignment constitutes a component of the dysplastic phenotype is an area worthy of more appreciation. The current treatment modalities primarily consider dysplasia as a bony deformity at which treatment is targeted. Future studies will have to look into the influence of physiotherapy and conservative treatment on PT [13]. Furthermore, there may be a role of considering PT in surgical treatment.

Given that the investigated patient cohort consisted exclusively of patients with symptomatic hip dysplasia, the question arises whether the observed lumbopelvic paradox may represent the tipping point for developing a symptomatic condition. To address this specific research question, an extensive analysis of a healthy cohort would be required. However, this would involve a difficult methodology due to radiation exposure.

This study bares several limitations that should be considered. First, it is acknowledged in the discussion that the degree of PT is associated with the extent of observed acetabular retroversion. In other words, the degree to which the observed acetabular version and thereby its associated acetabular phenotype are influenced by the present PT cannot be conclusively determined by this study. Another limitation of the study is that the lateral pelvic radiograph was taken in the standing position, while the AP pelvic radiograph was taken in the lying position. Due to the fact that the AP pelvic radiograph taken in the supine position represents the gold standard and the reference values available for assessment are based on supine x‐ray images, conducting pelvic x‐rays in the standing position was not justifiable and the modality was correspondingly accepted to determine the phenotype of hip dysplasia as generally agreed upon [4].

It is known that PT varies depending on body position. In addition to the variation in PT, the extent of displayed retroversion may also vary due to the functional pelvic position. Nevertheless, the same radiographic modalities were applied to all patients, maintaining the validity of the relationships between the determined values. Only the absolute values presented here should be critically appreciated due to this factor [8]. Consequently, it would be of significant interest to investigate the range of PT in relation to acetabular orientation during weight‐bearing and supine positions [8]. Additionally, analyzing the dynamic range of PT during gait in correlation with acetabular phenotype would be of interest, given that weight‐bearing activities exert considerable forces on an unstable hip joint [25]. Due to limitations in radiation exposure, we were unable to address these questions. Therefore, future studies incorporating camera‐assisted gait analysis would be of great importance.

## CONCLUSION

The results of this study reveal a paradox in the hip–spine association in hip dysplasia. Contrary to previous theories, PT seems to constitute a component of the corresponding phenotype of dysplastic pathology rather than functioning as a compensatory tilt.

## AUTHOR CONTRIBUTIONS

**Marco Haertlé**: Formal analysis; visualization; writing—original draft; funding acquisition. **Malte Lübbecke**: Data curation; review and editing. **Nils Becker**: Data curation; review and editing; funding acquisition. **Henning Windhagen**: Writing—review and editing; funding acquisition. **Quentin Karisch**: Data curation; review and editing. **Sufian S. Ahmad**: Writing—review and editing; supervision; funding acquisition.

## CONFLICT OF INTEREST STATEMENT

The authors declare no conflicts of interest.

## ETHICS STATEMENT

The study was conducted in accordance with the Declaration of Helsinki and approved by the local Ethics Committee of the Hannover Medical School (code: 11329_BO_K_2024 date: 04.04.2024). Investigation was performed at Hannover Medical School, Department of Orthopaedic Surgery, Anna‐von‐Borriesstr. 1‐7, 30625 Hannover, Germany.

## Data Availability

The data sets utilized in this study can be obtained from the corresponding author upon request.
